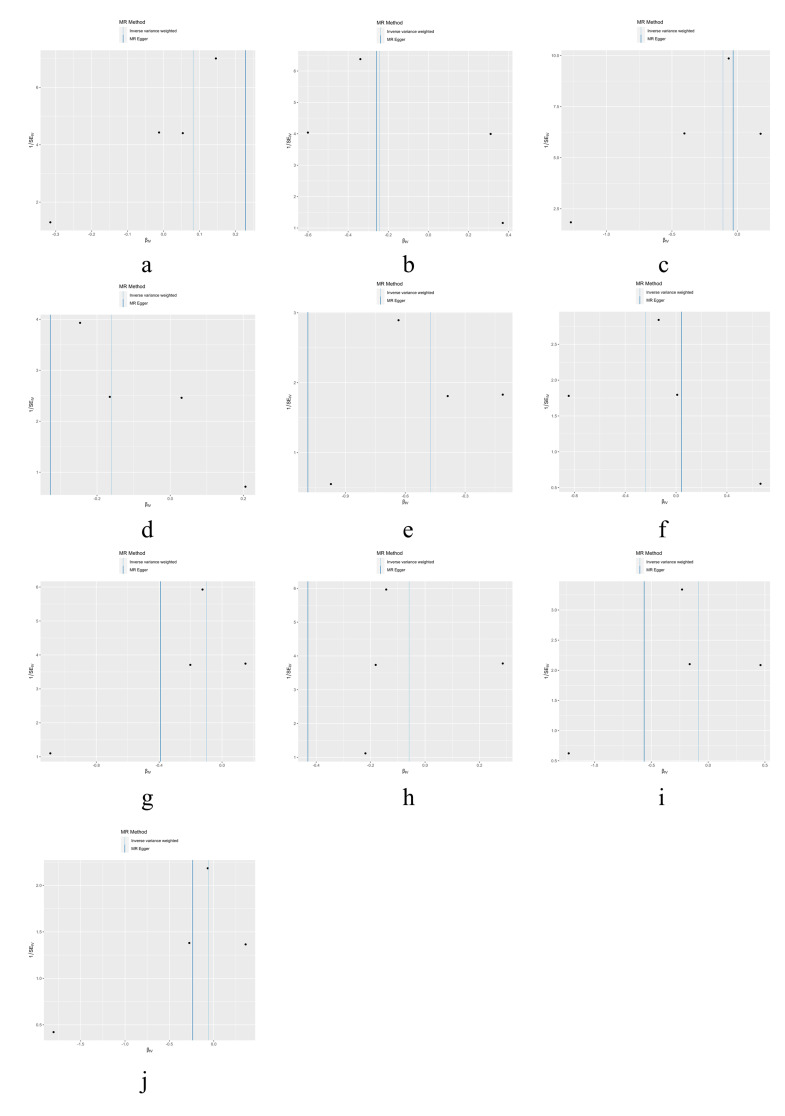# Gene association analysis to determine the causal relationship between immune-mediated inflammatory diseases and frozen shoulder

**DOI:** 10.1097/MD.0000000000038055

**Published:** 2024-05-10

**Authors:** Yuhang Zhou, Xiuping Yin, Chenyu Wang, Donglin Yu

**Affiliations:** a Binzhou Medical University, Yantai, China; b Experimental Research Center, China Academy of Chinese Medical Sciences, Beijing, China.

**Keywords:** frozen shoulder, genome-wide association studies, immune-mediated inflammatory diseases, Mendelian randomization, relationship

## Abstract

Multiple studies have indicated a potential correlation between immune-mediated inflammatory diseases (IMIDs) and Frozen shoulder (FS). To explore the genetic causal relationship between IMIDs and FS using 2-sample Mendelian randomization (MR) analysis. Genome-wide association study (GWAS) summary data for FS were obtained from Green’s study, while data for 10 IMIDs were sourced from the FinnGen Consortium. The MR analysis was performed using inverse variance weighting, MR Egger, and weighted median methods. IVW, as the primary MR analysis technique, was complemented with other sensitivity analyses to validate the robustness of the results. Additionally, reverse MR analysis was further conducted to investigate the presence of reverse causal relationships. In the forward MR analysis, genetically determined 4 IMIDs are causally associated with FS: rheumatoid arthritis (odds ratio [OR] (95% confidence interval [95% CI]) = 1.05 [1.02–1.09], *P* < .01); type 1 diabetes (OR [95% CI] = 1.06 [1.03–1.09], *P* < .01); hypothyroidism (OR [95% CI] = 1.07 [1.01–1.14], *P* = .02); and Celiac disease (OR [95% CI] = 1.02 [1.01–1.04], *P* = .01). However, no causal relationship was found between 6 IMIDs (autoimmune hyperthyroidism, Crohn disease, ulcerative colitis, psoriasis, sicca syndrome and systemic lupus erythematosus) and FS. Sensitivity analyses did not detect any heterogeneity or horizontal pleiotropy. In the reverse MR analysis, no causal relationship was observed between FS and IMIDs. In conclusion, this MR study suggests a potential causal relationship between rheumatoid arthritis, type 1 diabetes, hypothyroidism, and Celiac disease in the onset and development of FS. Nevertheless, more basic and clinical research will be needed in the future to support our findings.

## 1. Introduction

Frozen shoulder (FS) is a prevalent fibroproliferative condition characterized by a gradual decline in shoulder joint mobility and persistent pain.^[1]^ The lifetime prevalence of FS is estimated to be 2% to 5%, and it has been observed to affect the other shoulder within 5 years.^[2–5]^ Although FS is generally considered a self-limiting disease, symptoms such as stiffness and pain may persist in 20% to 50% of patients.^[6,7]^ The onset of FS is a complex pathological process that arises from the interaction of multiple etiological factors, involving high expression of TGF-ß to induce various cellular fibrotic responses^[8]^ and production of IL-17A from T cells causing greater fibrotic and inflammatory responses.^[9]^

These mechanisms highlight the immune-inflammatory nature of FS and its association with immune-inflammatory diseases (IMIDs). Moreover, current epidemiological studies indicate a relationship between FS and IMIDs such as thyroid dysfunction^[10,11]^ and type 1 diabetes (T1D).^[12]^ IMIDs are characterized by the immune system mistakenly attacking the body’s own tissues and organs. Autoimmune diseases, a subset of IMIDs, involve immune system dysregulation, the presence of self-antigens, inflammatory responses, and organ dysfunction.^[13,14]^ In this context, we speculate on a potential causal relationship between FS and other IMIDs such as rheumatoid arthritis (RA), psoriasis, and systemic lupus erythematosus (SLE), because these diseases can elicit inflammatory reactions around the joints, resulting in joint pain and functional limitations. However, currently, in clinical practice, there has been insufficient emphasis on analyzing the correlation between these diseases, and no relevant literature reports have been found.

In recent years, Mendelian randomization (MR) has emerged as a valuable method for investigating causal relationships between etiological factors and diseases, overcoming the limitations of observational studies and randomized controlled trials.^[15,16]^ MR utilizes genetic variants identified through genome-wide association studies (GWAS) as instrumental variables (IVs) to estimate the causal effects of modifiable exposures on outcomes.^[17]^ In MR studies, single nucleotide polymorphisms (SNPs) are used as IVs to estimate the causal associations between exposures and target outcomes. These SNPs adhere to the random distribution principle of genetic variation, their genotypes remain fixed, unaffected by the development of the disease, and confounding bias and reverse causality issues are mitigated.^[15]^

To address the limitations of clinical research and expand knowledge in this area, our study aims to employ a two-sample bidirectional MR approach using recently published GWAS data. The primary objective is to examine the existence of a direct causal relationship between 10 IMIDs and FS. By leveraging genetic instrumental variables, we can explore the potential causal links between these IMIDs and FS, shedding light on their interplay and providing insights into the underlying mechanisms.

## 2. Methods

### 2.1. Data source

We conducted a 2-sample bidirectional MR study using aggregated data from different GWASs on IMIDs and FS. To minimize potential confounding bias due to population stratification, we restricted the sample data to individuals of European ancestry from various regions worldwide.

The GWAS data for FS were derived from the study conducted by Green et al.^[18]^ To explore the biological mechanisms involved in FS and identify genetic variants associated with FS, the study performed a meta-analysis using FS data from the UK Biobank, which included 451,099 individuals and 15,184,371 SNPs. In this study, a linear mixed model was employed to test the association between each SNP and the outcome trait, including population structure as part of the model.^[19]^ Covariate adjustments were made for age, sex, study center, and genotyping array. A summary of GWAS data for FS can be downloaded from the IEU OpenGWAS repository at https://gwas.mrcieu.ac.uk/.

The GWAS datasets for different IMIDs were sourced from FinnGen. FinnGen is a large public-private partnership aimed at collecting and analyzing genomic and health data from 500,000 participants in the Finnish Biobank. SNPs were analyzed using a mixed-model logistic regression, adjusting for gender, age, 10 principal components, genetic relatedness, and genotyping batch. Detailed information about FinnGen can be found on its official website (https://www.finngen.fi/en). Table S1, Supplemental Digital Content, http://links.lww.com/MD/M441 provides a comprehensive list of the data sources and definitions.

This study obtained approval from the ethics committees of each institution, and all participants provided written informed consent.

### 2.2. Instrumental variable selection

Genetic variations were used as IVs to estimate causal relationships in MR analysis. If the following conditions were met, MR analysis can provide unbiased estimates of the causal relationship between IMIDs and FS: the selected IVs are exclusively associated with the 10 IMIDs; the IVs for the exposure are independent of any confounding factors; the IVs only affect FS through the 10 IMIDs (Figure S1, Supplemental Digital Content, http://links.lww.com/MD/M432). SNPs associated with each IMID were selected as potential IVs when they reached a genome-wide significance threshold of *P* < 5.0 × 10^−8^. To ensure independence among the genetic variations used as IVs, we set the linkage disequilibrium threshold for grouping at *R*^2^ < 0.001, with a window size of 1000 kb. To ensure accuracy and consistency in selecting SNPs as IVs across different analyses, if a variant was missing from the GWAS analysis, proxy SNPs were not substituted. The minimum allele frequency (MAF) was set to 0.001. In addition, we harmonized the effect alleles in both the exposure and outcome datasets, excluding all SNPs with palindromic effects. Weak IVs, which were IVs that were not strongly correlated with the exposure factor, had a poor performance in explaining the genetic variation of the exposure. To quantify IV strength, we utilize the *F*-statistic, where an *F*-statistic > 10 is considered to indicate sufficient instrument strength. The formula for calculating the *F*-statistic can be expressed as follows: *F* = ((*N* − K − 1)/*K*)*R^2^/(1 − *R*^2^). Where *R*^2^ represents the proportion of variance in the exposure explained by the SNP, *N* is the sample size, and *K* is the count of SNPs. The formula for *R*^2^ can be expressed as follows: *R*^2^ = 2 × MAF × (1 − MAF) × β^2^. Where MAF referd to the minor allele frequency and β represented the effect size of the exposure.^[20]^ In the absence of MAF, *R*^2^ was replaced with the formula *R*^2^ = β^2^/(β^2^ + SE^2^*N).^[21]^

### 2.3. Statistical analysis

In the MR analysis, we employed the multiplicative random-effects Inverse variance weighted (IVW), weighted median, and MR Egger methods. MR-Egger quantifies directional pleiotropy and provides an unbiased estimate even with pleiotropic effects, but it’s sensitive to outliers and less efficient. The weighted median is robust to outliers and provides an unbiased estimate even when up to half of the SNPs violate instrumental variable assumptions, although it may be less efficient. IVW calculates a weighted mean of individual variant effects on the outcome, assuming uncorrelated genetic variants, and offers optimal statistical power. In comparison to the fixed-effects IVW, the multiplicative random-effects IVW provided robust results in the presence of heterogeneity in instrument selection, with more conservative and realistic parameter estimates.^[22]^ Therefore, we considered the results of the IVW method as the primary outcome of the MR analysis. To enhance the robustness of our conclusions, we required that the IVW results be significant and that the results of the weighted median and MR-Egger methods be directionally consistent with the IVW results.

After the MR analysis, we assessed the heterogeneity of the effects of IMID-related SNPs on FS using Cochran’s *Q* test.^[23]^ In case of significant heterogeneity (*P* < .05), we employed the MR-PRESSO method to remove the IVs exhibiting heterogeneity from the analysis and conducted MR analysis again on the remaining IVs that were not identified as heterogeneous.^[24]^ We utilized MR-Egger regression to assess potential directional pleiotropy, where a value close to zero with a *P* value > .05 for the intercept term indicates the absence of horizontal pleiotropy.^[25]^ Using the PhenoScanner database (https://gwas.mrcieu.ac.uk/), we manually screened and omitted SNPs that were associated with confounding factors. Additionally, we performed leave-one-out analysis, forest plots, and funnel plots to assess the reliability and pleiotropy of the estimates. Scatter plots were generated to visualize the magnitude of the estimated effects.

In the analysis of reverse causality, we repeated the above methods and used a set of SNPs associated with FS to analyze the causal effects on the IMIDs.

All statistical analyses in this study were performed using the “TwoSampleMR” package in R software (version 4.1.0). All presented *P* values are 2-sided, and statistical significance was set at a level of 5%. We followed the recommendations of “STROBE-MR: Guidelines for Strengthening the Reporting of Mendelian Randomization Studies in Observational Research” to report our results.^[26]^

## 3. Results

### 3.1. Selection of instrumental variables

Based on established quality control criteria, SNPs associated with IMIDs were selected as IVs as follows: 12 SNPs for RA, 12 SNPs for T1D, 23 SNPs for Hypothyroidism, 10 SNPs for celiac disease (CeD), 6 SNPs for autoimmune hyperthyroidism (AIH), 4 SNPs for Crohn disease (CD), 5 SNPs for ulcerative colitis (UC), 9 SNPs for psoriasis, 2 SNPs for sicca syndrome (SS), and 3 SNPs for SLE. The *F* statistics for these IVs were greater than 10 (range: 11–36,518), indicating sufficient strength for the MR analysis. Detailed information on the SNPs as IVs is provided in Table S2, Supplemental Digital Content, http://links.lww.com/MD/M442.

### 3.2. Causal effects of IMIDs on FS

**Figure 1.**
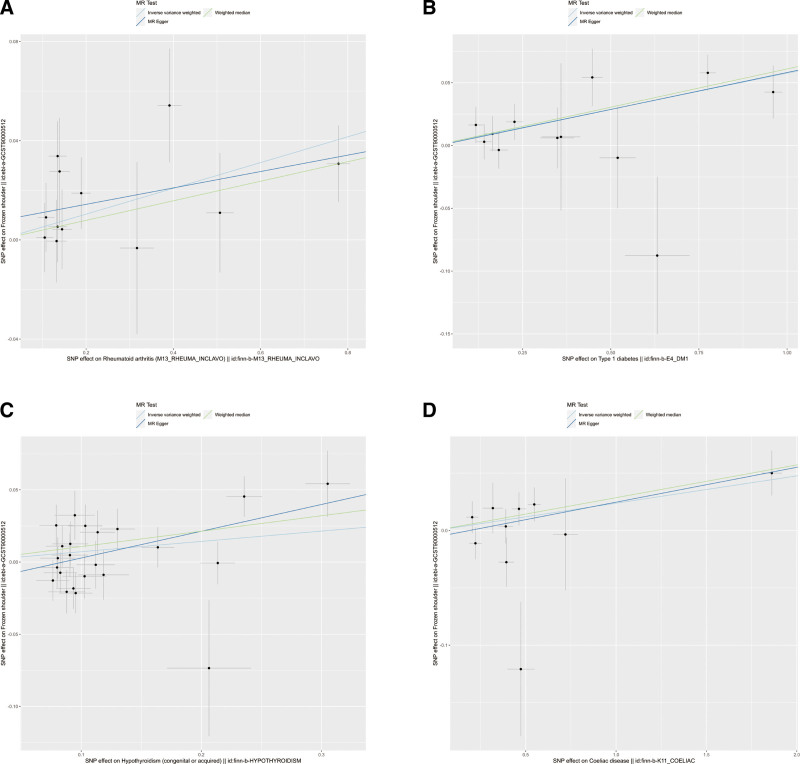
The scatter plots for the causal effect of IMIDs on frozen shoulder. (A) Rheumatoid arthritis on the frozen shoulder, (B) type 1 diabetes on the frozen shoulder, (C) hypothyroidism on the frozen shoulder, and (D) Coeliac disease on the frozen shoulder. IMIDs = immune-mediated inflammatory diseases.

**Figure 2.**
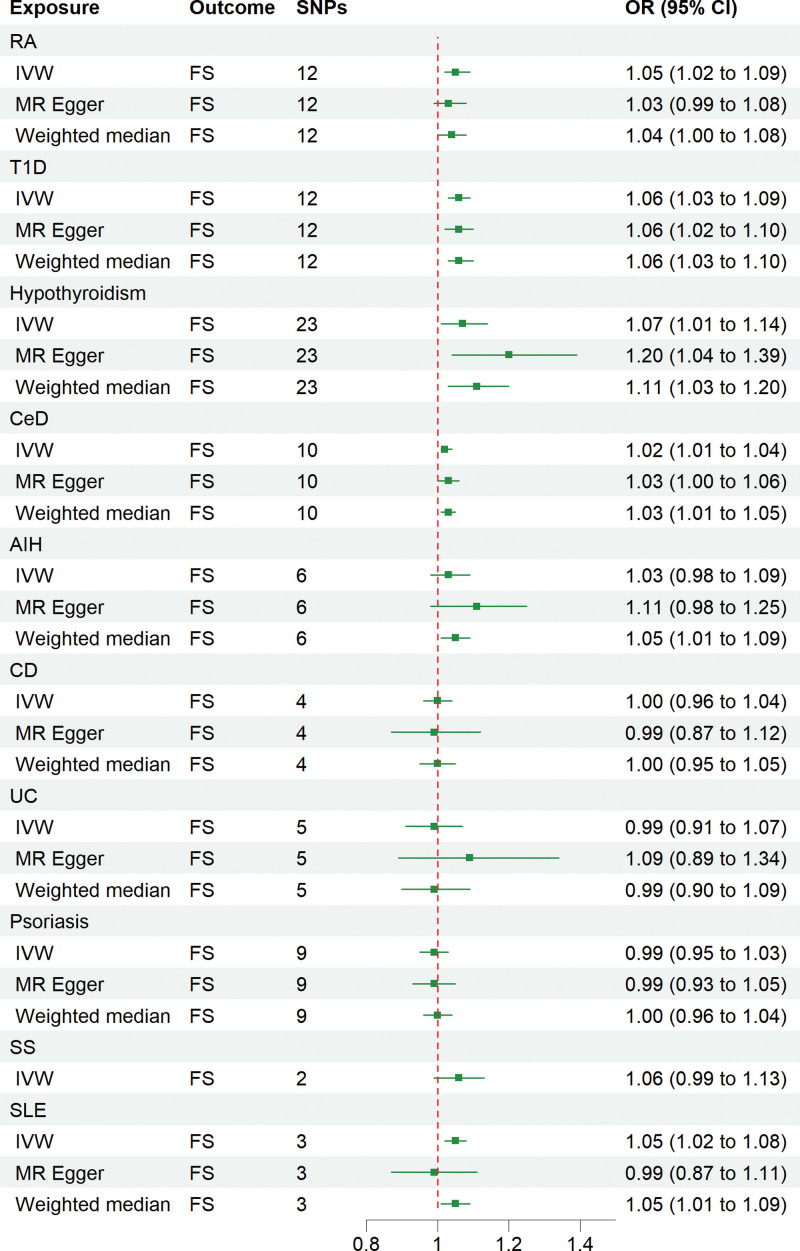
Forest plots of MR study using genetically predicted IMIDs with frozen shoulder. IVW, MR-Egger, and weighted median were used in this study. AIH = autoimmune hyperthyroidism, CD = Crohn disease, CeD = Coeliac disease, IMIDs = immune-mediated inflammatory diseases, IVW = inverse variance weighted, MR = Mendelian randomization, RA = rheumatoid arthritis, SNPs = single nucleotide polymorphisms, SLE = systemic lupus erythematosus, SS = sicca syndrome, T1D = type 1 diabetes, UC = ulcerative colitis.

The IVW, MR-Egger, and weighted median methods were used to assess the causal relationship between 10 IMIDs and the risk of FS. The results supported a causal relationship between genetic susceptibility to RA, T1D, Hypothyroidism, and CeD and an increased risk of FS. The IVW analysis revealed that there was a higher risk association between genetically predicted RA and FS (OR = 1.05, 95% CI: 1.02-1.09, *P* < .01), T1D and FS (OR = 1.06, 95% CI: 1.03-1.09, *P* < .01), hypothyroidism and FS (OR = 1.07, 95% CI: 1.01-1.14, *P* = .02), and CeD and FS (OR = 1.02, 95% CI: 1.01-1.04, *P* = .01) (Figs. 1 and 2). Similar results were observed in the MR-Egger and weighted median analyses, supporting the robustness of the inferred causal relationships between RA, T1D, hypothyroidism, CeD, and FS. However, no causal relationships were found between AIH, CD, UC, psoriasis, SS, SLE, and FS in this study.

The results of heterogeneity and horizontal pleiotropy were presented in Table 1. The MR-Egger regression showed no evidence of horizontal pleiotropy for any of the exposures. In Cochran’s *Q* test, all *P* values of the *Q* statistic for causal analyses were above 0.05, indicating the absence of heterogeneity among the IVs. Leave-one-out analysis further confirmed the stability of the results, as shown in Figure S4, Supplemental Digital Content, http://links.lww.com/MD/M435. Scatter plots for the MR analysis can be found in Figure 1 and Figure S2, Supplemental Digital Content, http://links.lww.com/MD/M433, funnel plots in Figure S5, Supplemental Digital Content, http://links.lww.com/MD/M436, and forest plots in Figure S3, Supplemental Digital Content, http://links.lww.com/MD/M434. Therefore, we considered the results from the IVW analysis to be reliable.

**Table 1:** Heterogeneity and pleiotropy analysis in forward MR analysis.

Exposure	MR method	Cochran *Q* statistic	Egger intercept	Heterogeneity (*P* value)	Pleiotropy (*P* value)
RA	MR Egger	7.45	0.008	.682	.309
	IVW	8.60		.658	
T1D	MR Egger	9.64	−0.001	.473	.935
	IVW	9.65		.562	
Hypothyroidism	MR Egger	30.49	−0.016	.083	.114
	IVW	34.44		.054	
CeD	MR Egger	10.60	−0.006	.225	.572
	IVW	11.06		.271	
AIH	MR Egger	11.52	−0.041	.211	.298
	IVW	15.63		.080	
CD	MR Egger	1.87	0.010	.393	.851
	IVW	1.91		.591	
UC	MR Egger	0.43	−0.020	.934	.365
	IVW	1.57		.815	
Psoriasis	MR Egger	8.49	0.001	.292	.949
	IVW	8.49		.387	
SS	IVW	2.08	NA	.149	NA
SLE	MR Egger	0.00	0.041	.965	.495
	IVW	1.03		.596	

AIH = autoimmune hyperthyroidism, CD = Crohn disease, CeD = Coeliac disease, IVW = inverse variance weighted, MR = Mendelian randomization, RA = rheumatoid arthritis, SLE = systemic lupus erythematosus, SS = sicca syndrome, T1D = type 1 diabetes, UC = ulcerative colitis.

### 3.3. Causal effects of FS on IMIDs

Finally, we conducted a reverse MR analysis to examine whether the genetic predisposition to FS has a causal effect on the IMIDs. We selected 4 SNPs (rs1042704, rs117999064, rs5777216, and rs62228068) that were associated with FS as IVs. Although some of the SNPs had *F* values less than 10, considering the limited number of SNPs, we chose to retain them (Table S3, Supplemental Digital Content, http://links.lww.com/MD/M443). However, the reverse MR analysis did not reveal any significant results, as shown in Table 2. Additionally, tests for heterogeneity and MR-Egger intercept did not indicate any notable deviations. Further details were provided in Table S4, Supplemental Digital Content, http://links.lww.com/MD/M444. Visualizations of the results for FS and IMIDs can be found in Figures S6 to S9, Supplemental Digital Content, http://links.lww.com/MD/M437, http://links.lww.com/MD/M438, http://links.lww.com/MD/M439, http://links.lww.com/MD/M440.

**Table 2:** MR results between IMIDs and frozen shoulder.

Outcome	MR methods	SNPs	OR (95% CI)	*P* value
RA	IVW	4	1.09 (0.88–1.34)	.43
	MR Egger	4	1.26 (0.67–2.35)	.55
	Weighted median	4	1.09 (0.87–1.37)	.43
T1D	IVW	4	0.78 (0.54–1.13)	.19
	MR Egger	4	0.77 (0.2–3.02)	.75
	Weighted median	4	0.73 (0.55–0.97)	.03
Hypothyroidism	IVW	4	0.9 (0.67–1.19)	.45
	MR Egger	4	0.97 (0.34–2.76)	.96
	Weighted median	4	0.94 (0.77–1.14)	.52
CeD	IVW	4	0.85 (0.59–1.23)	.39
	MR Egger	4	0.72 (0.24–2.2)	.62
	Weighted median	4	0.82 (0.55–1.22)	.33
AIH	IVW	4	0.62 (0.38–1.03)	.06
	MR Egger	4	0.34 (0.07–1.53)	.29
	Weighted median	4	0.61 (0.34–1.08)	.09
CD	IVW	4	0.79 (0.47–1.31)	.36
	MR Egger	4	1.04 (0.22–4.85)	.96
	Weighted median	4	0.88 (0.48–1.6)	.67
UC	IVW	4	0.91 (0.71–1.16)	.43
	MR Egger	4	0.68 (0.32–1.42)	.41
	Weighted median	4	0.88 (0.67–1.17)	.38
Psoriasis	IVW	4	0.94 (0.74–1.2)	.64
	MR Egger	4	0.65 (0.31–1.35)	.37
	Weighted median	4	0.87 (0.66–1.14)	.3
SS	IVW	4	0.92 (0.59–1.42)	.7
	MR Egger	4	0.57 (0.15–2.11)	.49
	Weighted median	4	0.82 (0.5–1.37)	.45
SLE	IVW	4	0.94 (0.49–1.84)	.87
	MR Egger	4	0.79 (0.11–5.84)	.84
	Weighted median	4	0.93 (0.45–1.91)	.84

AIH = autoimmune hyperthyroidism, CD = Crohn disease, CeD = Coeliac disease, IMIDs = immune-mediated inflammatory diseases, IVW = inverse variance weighted, MR = Mendelian randomization, RA = rheumatoid arthritis, SLE = systemic lupus erythematosus, SNPs = single nucleotide polymorphisms, SS = sicca syndrome, T1D = type 1 diabetes, UC = ulcerative colitis.

## 4. Discussion

To the best of our knowledge, this study represents the first large-scale 2-sample MR investigation examining the potential causal association between IMIDs and FS. The results of our analysis indicate a positive correlation between genetic predisposition to RA, T1D, hypothyroidism, and CeD and the risk of developing FS. However, our MR analysis did not provide evidence supporting a causal relationship between genetic susceptibility to AIH, CD, UC, Psoriasis, SS, and SLE, and the risk of FS. These findings were robust and consistent across various sensitivity analyses, further strengthening the reliability of our results.

FS is a disease that involves damage to the structures around the shoulder joint, resulting in shoulder pain and restricted movement. IMIDs, on the other hand, involve a diverse group of disorders characterized by immune dysregulation, leading to aberrant reactions of B cells and T cells against normal components of the host.^[14]^ The occurrence of FS has been associated with increased pro-inflammatory mediators, inflammatory response, activated fibroblasts, and heightened cytokine activity. These parallels in immune dysregulation,^[27,28]^ abnormal recognition of self-antigens,^[29,30]^ and inflammatory response^[31,32]^ are reminiscent of the characteristics observed in IMIDs. Additionally, the interplay of genetic and environmental factors^[33,34]^ observed in both FS and IMIDs further supports a potential connection between these conditions.

Previous studies have shown an association between T1D, hypothyroidism, and FS, which is consistence with our research. In T1D patients with a disease duration of more than 45 years, the lifetime prevalence of FS is 76%, significantly higher than the prevalence among non-diabetic individuals (14%).^[35]^ T1D patients have a 1.05 times higher risk of developing FS compared to non-T1D individuals.^[18]^ A cross-sectional study found that the chance of developing FS in patients with Hypothyroidism was 2.69 times higher than that of the normal population^[10]^ and this chance may increase to 5 times during the COVID-19 pandemic.^[36]^ Kumar et al^[37]^ proposed that diabetes and thyroid diseases are characterized by a pro-inflammatory state, elevated levels of inflammatory cytokines, and increased fibrosis, leading to an inflammatory response in the shoulder joint, promoting fibrosis in the muscles around the shoulder, and further increase the risk of developing FS.

Our study revealed an increased risk of FS associated with RA and CeD, which is an interesting finding not previously reported in the existing research. Several factors could explain this association. Firstly, elevated levels of IL-33, an alarm protein, have been discovered in FS tissues by Cher et al^[38]^ discovered elevated levels of IL-33 in FS tissues, indicating its alarm function. However, previous research has identified the release of similar alarm proteins in RA, indicating its role in signaling inflammation. Interestingly, previous research has also identified the release of similar alarm proteins in RA.^[39,40]^ This suggests a potential common pathway involving inflammatory signaling in both RA and FS. Secondly, a comparison of patients with remission RA and healthy shoulder joint capsules has shown the presence of the same subtype of macrophages. However, these macrophages were found to be reduced in the joint capsules of FS patients.^[9,41]^ This finding suggests that there may be shared pathogenic mechanisms between RA and FS, possibly involving macrophage-related processes. CeD is indeed a chronic autoimmune disorder characterized by an abnormal immune response to gluten proteins found in wheat and related grains. This immune reaction leads to damage to the small intestinal mucosa.^[42]^ While CeD primarily affects the gastrointestinal tract, there is evidence to suggest that the inflammatory response triggered by CeD may have systemic effects and affect other tissues and joints. Speculatively, the immune reaction in CeD could potentially initiate an inflammatory response that extends beyond the intestines and subsequently affects the joints, including the shoulder joint. This hypothesis is supported by the association we observed between CeD and FS in our study. Therefore, it is crucial for clinicians to be vigilant and consider the possibility of FS in patients with CeD, in addition to those with T1D, RA, and hypothyroidism, as mentioned earlier. Recognizing the potential association between these conditions and FS can aid in early detection, prompt management, and improved patient outcomes. Further research is needed to fully understand the underlying mechanisms linking CeD and FS. By gaining a better understanding of the immune and inflammatory processes involved, we can potentially develop more targeted approaches for the prevention and treatment of FS in individuals with CeD.

It is important to acknowledge that the association between AIH and FS remains inconclusive. Different studies have reported conflicting findings regarding this relationship. One study did not find a significant correlation between AIH and FS,^[36]^ but another study suggested a 1.22 times higher risk of developing FS in AIH patients during a 7-year follow-up.^[11]^ These discrepancies could be attributed to factors such as unmeasured confounders and variations in sample sizes among different studies. Our analysis did not provide evidence supporting a causal relationship between AIH and genetic susceptibility to FS. This suggests that there may not be a direct causal link between AIH and the development of FS. Although elevated levels of thyroid hormones can contribute to an overactive immune system and the development of immune-related disorders, it is possible that the association between thyroid diseases and FS may not be statistically significant. Furthermore, the contradictory nature of research findings may stem from differences in methodologies, sample selection, and statistical analyses among studies. Unmeasured confounding factors, such as lifestyle and genetic influences, could also impact the true association between AIH and FS. Additionally, variations in sample sizes across different studies, with some having smaller sample sizes, may contribute to the instability and inconsistency of results, resulting in a lack of consensus in conclusions. Further research is necessary to investigate the potential mechanisms underlying this relationship and to clarify the role of thyroid hormones in FS development.

However, our study did not find any association between the remaining IMIDs and FS and no studies have been found regarding the potential relationship between CD, UC, Psoriasis, SS, SLE, and FS. These diseases primarily affect the digestive tract (CD and UC) or the skin (Psoriasis, SS, and SLE), and their pathogenic mechanisms may differ from FS. In addition, there may be significant differences in the pathogenic mechanisms of different IMIDs, leading to diverse clinical manifestations and involvement of different body parts. Further research is necessary to better understand the specific relationship between these diseases and FS in the future.

The role of IMIDs in the occurrence and progression of FS is complex and potentially interactive. Although our MR analysis was able to exclude the influence of their interactions and assess their relationship from a genetic perspective, our study has inevitable limitations. Firstly, the results of this study are the product of statistical analysis, and the correlation between some IMIDs and FS has not been reported, lacking theoretical support. In the future, more basic and clinical research is needed to support our findings. Secondly, we were unable to perform subgroup analyses, such as age and gender, as our study utilized summary-level data of FS rather than individual-level data. Future research investigating the correlation between IMIDs and FS should consider the overall impact of these factors. Finally, we only utilized genetic data from individuals of European ancestry, limiting the generalizability of our findings.

In conclusion, our study underscores RA, T1D, hypothyroidism, and CeD as significant FS risk factors. Orthopedic doctors should prioritize patient screening and monitoring for early FS detection. Lifestyle modifications, alongside tailored treatment approaches, may aid in prevention. However, further research is crucial to validate and extend these findings for better patient care.

## Author contributions

**Conceptualization:** Yuhang Zhou.

**Data curation:** Xiuping Yin.

**Funding acquisition:** Donglin Yu.

**Investigation:** Chenyu Wang.

**Methodology:** Xiuping Yin.

**Resources:** Donglin Yu.

**Software:** Yuhang Zhou, Chenyu Wang.

**Supervision:** Donglin Yu.

**Visualization:** Xiuping Yin, Chenyu Wang.

**Writing – original draft:** Yuhang Zhou.

**Writing – review & editing:** Yuhang Zhou.

## Supplementary Material



**Figure:**
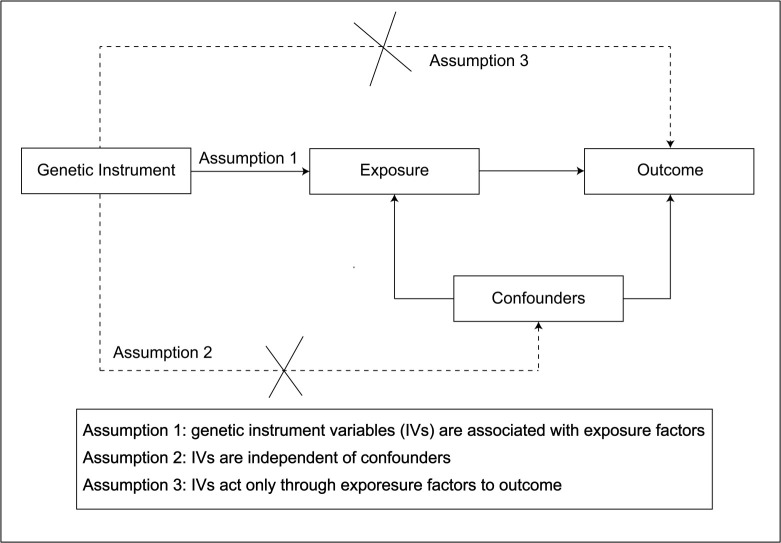




**Figure:**
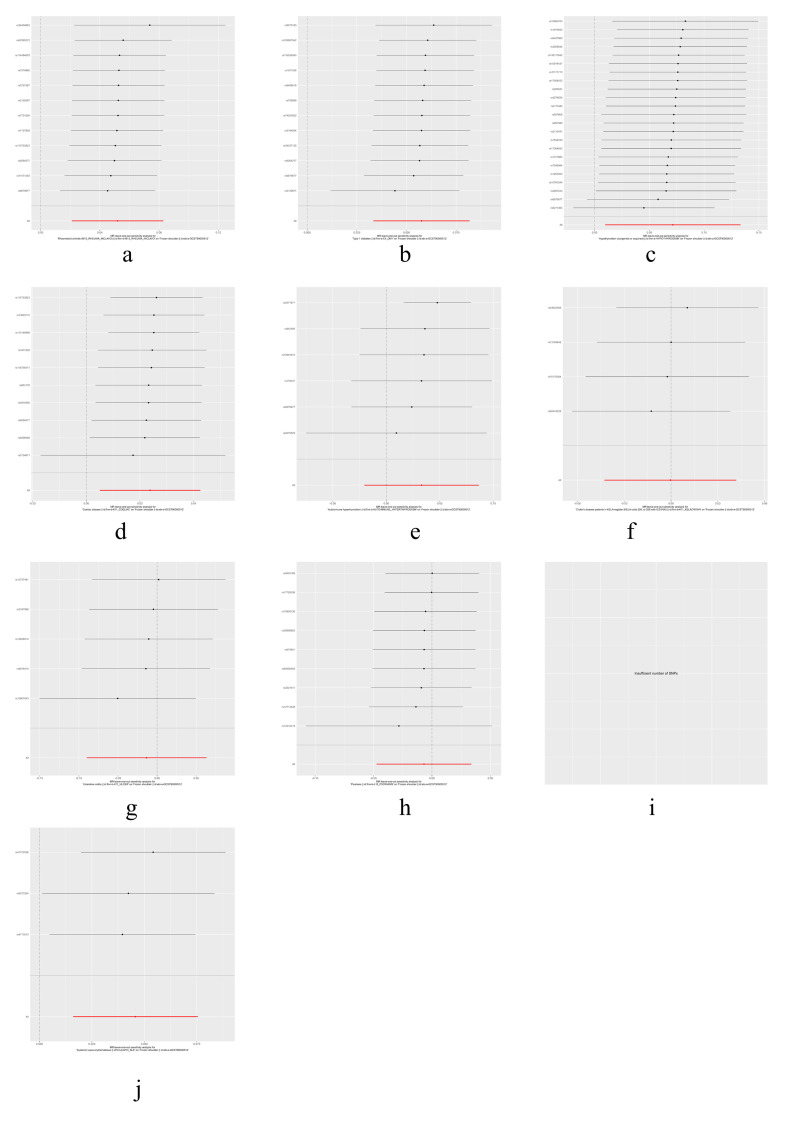


**Figure:**
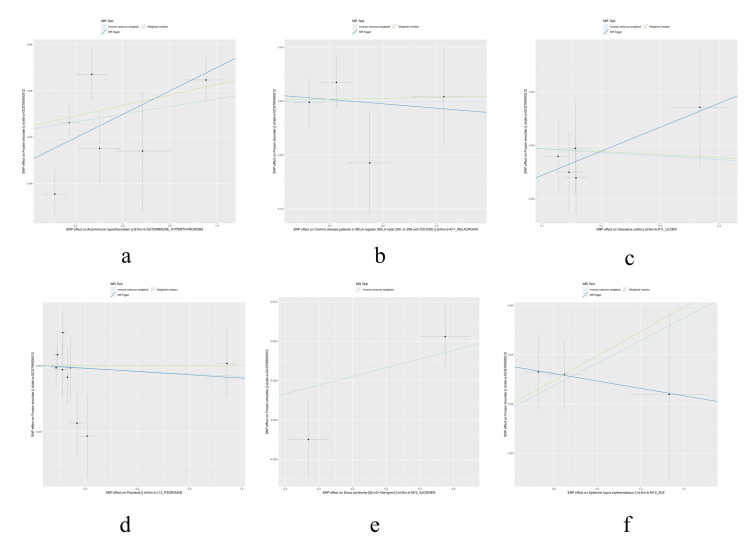


**Figure:**
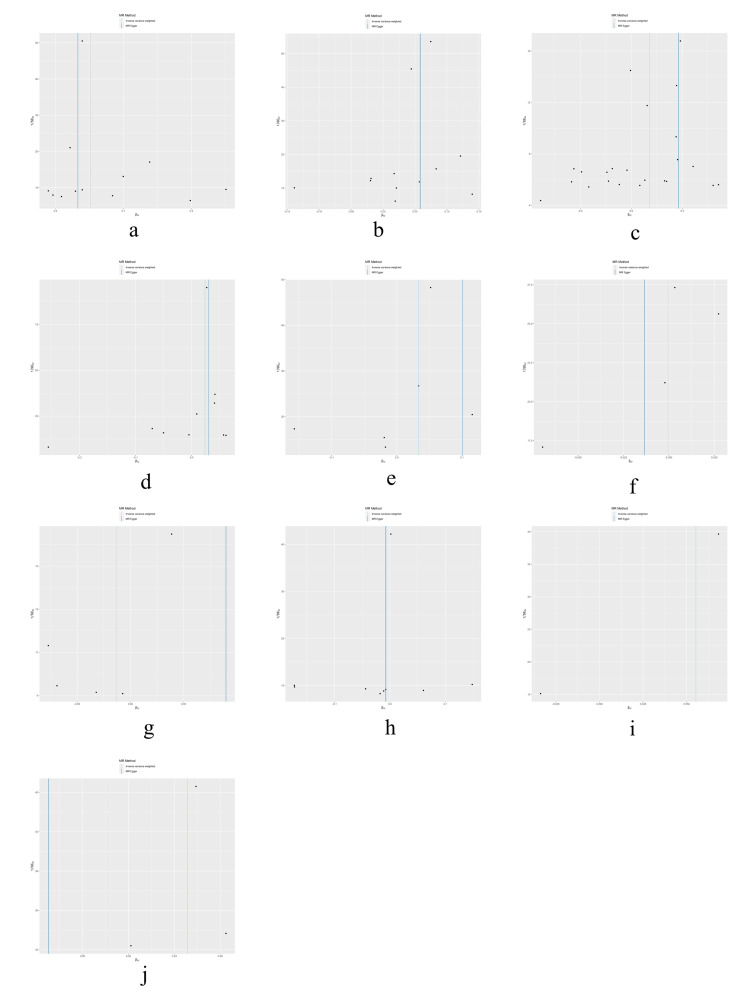


**Figure:**
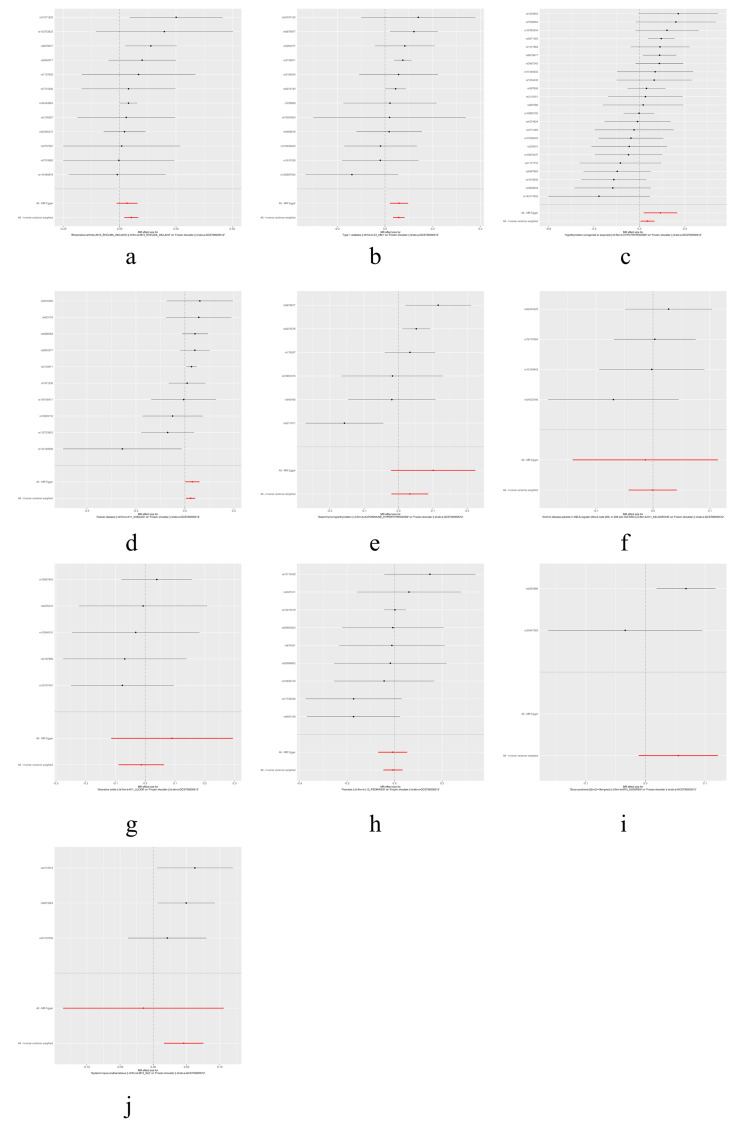






**Figure:**
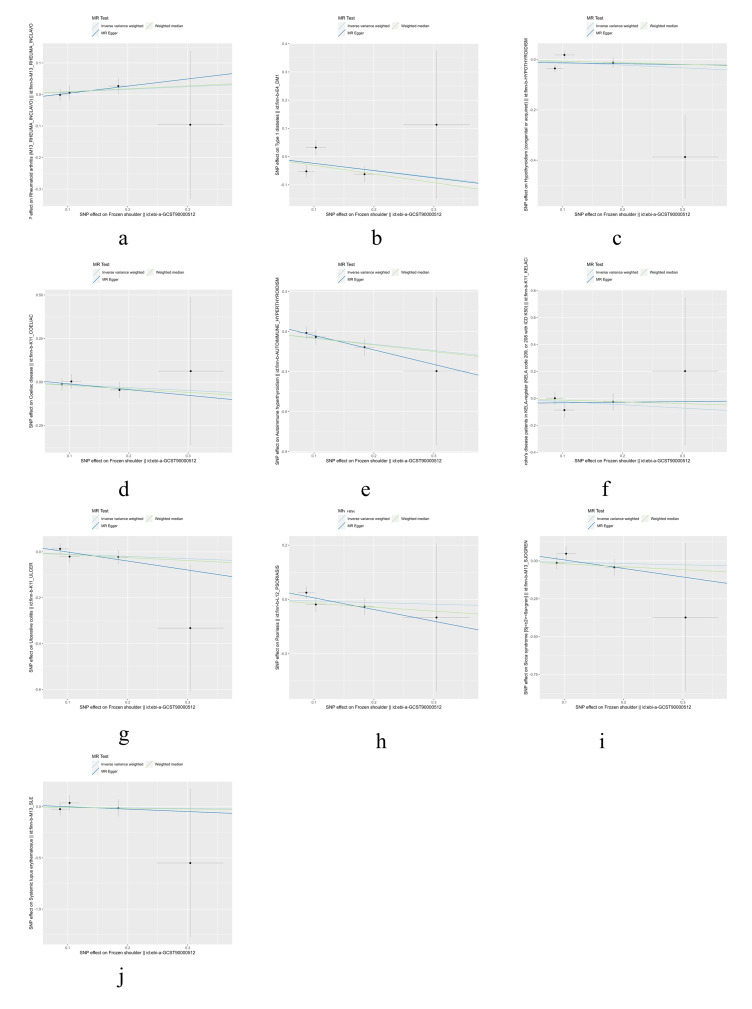


**Figure:**
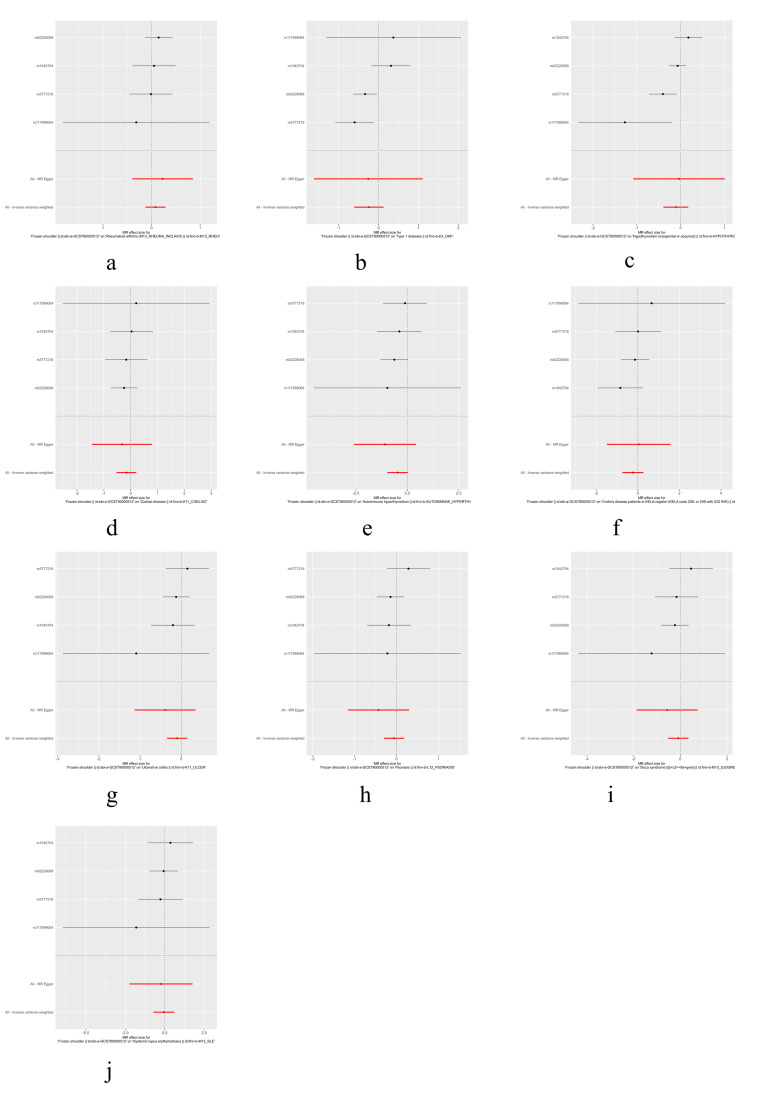


**Figure:**
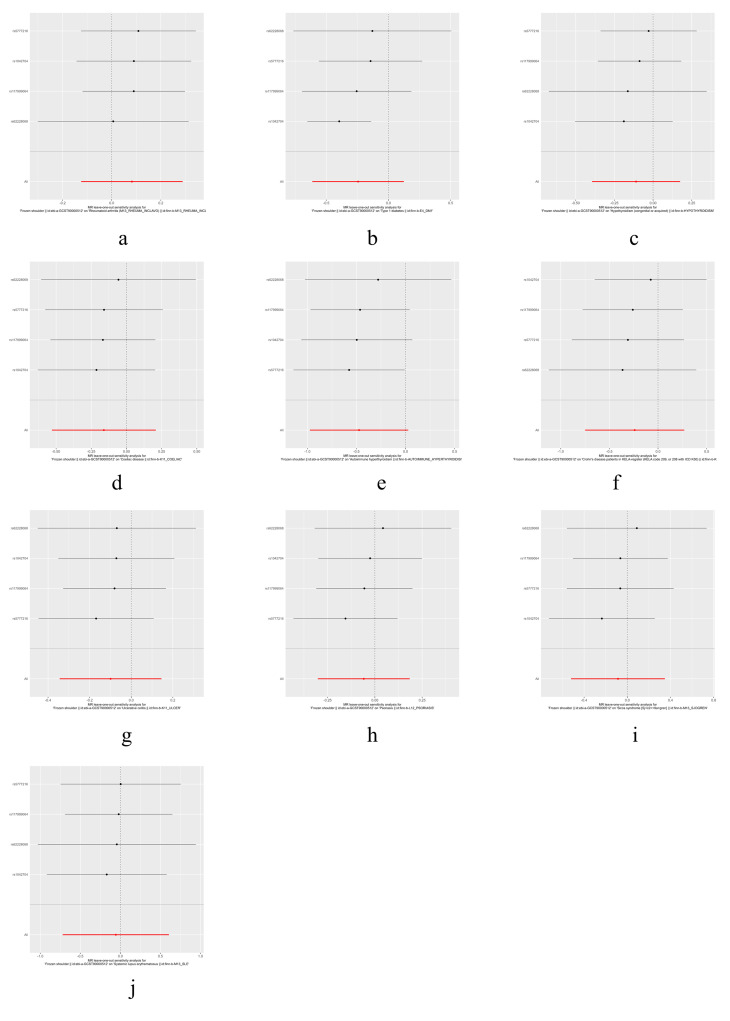


**Figure:**