# Supplementary material for: Gene association analysis to determine the causal relationship between immune-mediated inflammatory diseases and frozen shoulder
Source: Medicine (Baltimore). 2024 May 10;103(19):e38055. doi: 10.1097/MD.0000000000038055 (PMC11081594; doi:10.1097/MD.0000000000038055)
Supplement: Supplementary file 1 [file medi-103-e38055-s001.docx]

**Supplementary Table 1** | GWAS summary statistics: source and description

| **ID** | **Phenotypes** | **Year** | **Data source** | **Population** | **Sample size(cases)** |
| --- | --- | --- | --- | --- | --- |
| finn-b-M13_RHEUMA_INCLAVO | RA | 2021 | FinnGen | European | 218792 |
| finn-b-E4_DM1 | T1D | 2021 | FinnGen | European | 189113 |
| finn-b-HYPOTHYROIDISM | Hypothyroidism | 2021 | FinnGen | European | 86169 |
| finn-b-K11_COELIAC | CeD | 2021 | FinnGen | European | 212937 |
| finn-b-AUTOIMMUNE_HYPERTHYROIDISM | AIH | 2021 | FinnGen | European | 173938 |
| finn-b-K11_KELACROHN | CD | 2021 | FinnGen | European | 218792 |
| finn-b-K11_ULCER | UC | 2021 | FinnGen | European | 214620 |
| finn-b-L12_PSORIASIS | Psoriasis | 2021 | FinnGen | European | 216752 |
| finn-b-M13_SJOGREN | SS | 2021 | FinnGen | European | 214435 |
| finn-b-M13_SLE | SLE | 2021 | FinnGen | European | 213683 |
| ebi-a-GCST90000512 | FS | 2021 | Green HD et al | European | 451099 |

RA, Rheumatoid arthritis; T1D, Type 1 diabetes; CeD, Coeliac disease; AIH, Autoimmune hyperthyroidism; CD, Crohn's disease; UC, Ulcerative colitis; SS, Sicca syndrome; SLE, Systemic lupus erythematosus; FS, Frozen shoulder.
