# Supplementary material for: Gene association analysis to determine the causal relationship between immune-mediated inflammatory diseases and frozen shoulder
Source: Medicine (Baltimore). 2024 May 10;103(19):e38055. doi: 10.1097/MD.0000000000038055 (PMC11081594; doi:10.1097/MD.0000000000038055)
Supplement: Supplementary file 2 [file medi-103-e38055-s003.docx]

**Supplementary Table 2** | Characteristics of SNPs associated with IMIDs.

| RA | | | | | | | | | |
| --- | --- | --- | --- | --- | --- | --- | --- | --- | --- |
| SNP | EA | Position | EAF | BETA | SE | P | N | R^2^ | F |
| rs112733823 | T | 30775277 | 0.22 | 0.139 | 0.023 | 2.23E-09 | 218792 | 6.58E-03 | 121 |
| rs114484678 | C | 32215057 | 0.07 | 0.317 | 0.039 | 5.89E-16 | 218792 | 1.23E-02 | 226 |
| rs11571293 | T | 204717713 | 0.32 | -0.135 | 0.021 | 5.46E-11 | 218792 | 7.84E-03 | 144 |
| rs2922996a | C | 31337207 | 0.40 | 0.203 | 0.021 | 7.08E-23 | 218792 | 1.98E-02 | 368 |
| rs3093017a | G | 167541258 | 0.55 | -0.106 | 0.019 | 4.02E-08 | 218792 | 5.50E-03 | 101 |
| rs3129287 | T | 33089104 | 0.25 | -0.145 | 0.022 | 8.83E-11 | 218792 | 7.84E-03 | 144 |
| rs34434863 | G | 32559673 | 0.34 | 0.779 | 0.027 | 5.74E-188 | 218792 | 2.71E-01 | 6793 |
| rs34536443a | C | 10463118 | 0.03 | -0.321 | 0.057 | 1.61E-08 | 218792 | 6.06E-03 | 111 |
| rs3757387 | C | 128576086 | 0.42 | 0.105 | 0.019 | 4.82E-08 | 218792 | 5.39E-03 | 99 |
| rs62395272 | T | 31394424 | 0.11 | 0.507 | 0.032 | 5.30E-58 | 218792 | 5.04E-02 | 968 |
| rs6679677 | A | 114303808 | 0.15 | 0.391 | 0.028 | 6.93E-46 | 218792 | 3.84E-02 | 728 |
| rs7137828 | T | 111932800 | 0.59 | -0.108 | 0.019 | 2.10E-08 | 218792 | 5.67E-03 | 104 |
| rs7574865 | G | 191964633 | 0.77 | -0.132 | 0.023 | 4.51E-09 | 218792 | 6.24E-03 | 115 |
| rs7731626 | A | 55444683 | 0.28 | -0.135 | 0.021 | 2.54E-10 | 218792 | 7.30E-03 | 134 |
| rs9264277 | C | 31224667 | 0.73 | 0.189 | 0.022 | 1.67E-17 | 218792 | 1.41E-02 | 260 |
| T1D | | | | | | | | | |
| SNP | EA | Position | EAF | BETA | SE | P | N | R^2^ | F |
| rs116039340 | T | 32849821 | 0.05 | -0.52 | 0.051 | 4.92E-24 | 189113 | 2.56E-02 | 414 |
| rs1611236 | A | 29748690 | 0.22 | -0.182 | 0.026 | 1.70E-12 | 189113 | 1.15E-02 | 184 |
| rs183697542 | T | 30846941 | 0.02 | -0.632 | 0.092 | 5.88E-12 | 189113 | 1.31E-02 | 209 |
| rs3129871 | C | 32406342 | 0.64 | 0.775 | 0.023 | 1.00E-200 | 189113 | 2.75E-01 | 5985 |
| rs3184504 | C | 111884608 | 0.59 | -0.165 | 0.021 | 8.97E-15 | 189113 | 1.31E-02 | 209 |
| rs34337125 | A | 101307703 | 0.42 | -0.117 | 0.021 | 3.51E-08 | 189113 | 6.68E-03 | 106 |
| rs41173a | G | 30424863 | 0.68 | -0.135 | 0.022 | 1.31E-09 | 189113 | 7.93E-03 | 126 |
| rs6679677 | A | 114303808 | 0.15 | 0.448 | 0.03 | 8.24E-52 | 189113 | 5.03E-02 | 835 |
| rs689a | T | 2182224 | 0.79 | 0.399 | 0.027 | 5.29E-48 | 189113 | 5.25E-02 | 872 |
| rs705699 | A | 56384804 | 0.39 | 0.141 | 0.022 | 5.24E-11 | 189113 | 9.41E-03 | 150 |
| rs74203920 | T | 45714294 | 0.04 | 0.359 | 0.055 | 6.63E-11 | 189113 | 9.23E-03 | 147 |
| rs9264277 | C | 31224667 | 0.73 | 0.227 | 0.024 | 2.73E-21 | 189113 | 2.03E-02 | 326 |
| rs9275183 | G | 32654502 | 0.22 | 0.961 | 0.026 | 1.00E-200 | 189113 | 3.15E-01 | 7258 |
| rs9348894a | T | 32732677 | 0.45 | -0.402 | 0.025 | 5.26E-59 | 189113 | 7.99E-02 | 1368 |
| rs9468618 | T | 29750776 | 0.06 | -0.348 | 0.048 | 5.55E-13 | 189113 | 1.28E-02 | 205 |
| Hypothyroidism | | | | | | | | | |
| SNP | EA | Position | EAF | BETA | SE | P | N | R^2^ | F |
| rs10760344 | T | 127026282 | 0.35 | 0.103 | 0.014 | 4.96E-13 | 86169 | 4.85E-03 | 18 |
| rs10974437 | G | 4285547 | 0.17 | -0.103 | 0.018 | 1.72E-08 | 86169 | 2.98E-03 | 11 |
| rs10983700 | C | 100537455 | 0.66 | 0.214 | 0.014 | 5.18E-50 | 86169 | 2.04E-02 | 78 |
| rs11171710 | A | 56368078 | 0.45 | -0.076 | 0.014 | 2.97E-08 | 86169 | 2.88E-03 | 11 |
| rs1203943 | C | 22596825 | 0.79 | 0.095 | 0.017 | 1.81E-08 | 86169 | 2.94E-03 | 11 |
| rs1317983 | C | 43806335 | 0.68 | 0.114 | 0.015 | 1.12E-14 | 86169 | 5.60E-03 | 21 |
| rs143117642 | A | 6131244 | 0.04 | -0.206 | 0.035 | 3.68E-09 | 86169 | 3.24E-03 | 12 |
| rs1534430 | T | 12644736 | 0.41 | -0.084 | 0.014 | 8.64E-10 | 86169 | 3.44E-03 | 13 |
| rs17008423 | T | 149624705 | 0.12 | -0.119 | 0.021 | 2.33E-08 | 86169 | 2.97E-03 | 11 |
| rs17364832 | G | 24786915 | 0.29 | 0.091 | 0.015 | 1.06E-09 | 86169 | 3.42E-03 | 13 |
| rs17786733a | A | 1410929 | 0.43 | 0.079 | 0.014 | 1.18E-08 | 86169 | 3.02E-03 | 11 |
| rs1915930 | T | 188100256 | 0.62 | 0.095 | 0.014 | 1.03E-11 | 86169 | 4.28E-03 | 16 |
| rs1993945a | T | 76518195 | 0.42 | 0.126 | 0.014 | 5.98E-20 | 86169 | 7.69E-03 | 29 |
| rs2110451 | A | 9931347 | 0.28 | 0.091 | 0.015 | 2.04E-09 | 86169 | 3.31E-03 | 12 |
| rs2111485 | G | 163110536 | 0.59 | 0.08 | 0.014 | 7.35E-09 | 86169 | 3.10E-03 | 12 |
| rs229531 | C | 37585736 | 0.40 | 0.083 | 0.014 | 2.38E-09 | 86169 | 3.29E-03 | 12 |
| rs2553610a | G | 133763567 | 0.47 | -0.078 | 0.014 | 1.03E-08 | 86169 | 3.02E-03 | 11 |
| rs2844542a | G | 31347274 | 0.34 | 0.109 | 0.015 | 2.28E-13 | 86169 | 5.32E-03 | 20 |
| rs3008034 | C | 166043862 | 0.26 | -0.088 | 0.015 | 1.19E-08 | 86169 | 2.98E-03 | 11 |
| rs3087243 | A | 204738919 | 0.33 | -0.13 | 0.015 | 3.11E-19 | 86169 | 7.45E-03 | 28 |
| rs4274624 | T | 191958656 | 0.76 | -0.112 | 0.016 | 2.59E-12 | 86169 | 4.52E-03 | 17 |
| rs597808 | G | 111973358 | 0.58 | -0.164 | 0.014 | 8.70E-33 | 86169 | 1.31E-02 | 50 |
| rs6679677 | A | 114303808 | 0.15 | 0.305 | 0.019 | 5.47E-60 | 86169 | 2.42E-02 | 93 |
| rs7599564 | G | 43509617 | 0.63 | 0.079 | 0.014 | 1.80E-08 | 86169 | 2.94E-03 | 11 |
| rs7754251a | C | 90989125 | 0.45 | 0.082 | 0.014 | 1.82E-09 | 86169 | 3.32E-03 | 12 |
| rs897586 | A | 128193294 | 0.32 | -0.081 | 0.015 | 3.83E-08 | 86169 | 2.80E-03 | 11 |
| rs9271365 | G | 32586794 | 0.37 | 0.236 | 0.015 | 3.40E-56 | 86169 | 2.59E-02 | 100 |
| rs9277457a | G | 33053167 | 0.19 | -0.118 | 0.017 | 9.50E-12 | 86169 | 4.34E-03 | 16 |
| rs9497965 | T | 148521292 | 0.34 | 0.094 | 0.014 | 8.44E-11 | 86169 | 3.92E-03 | 15 |
| CeD | | | | | | | | | |
| SNP | EA | Position | EAF | BETA | SE | P | N | R^2^ | F |
| rs112733823 | T | 30775277 | 0.22 | -0.393 | 0.043 | 5.88E-20 | 212937 | 5.25E-02 | 1179 |
| rs113149998 | A | 35190836 | 0.04 | 0.473 | 0.076 | 4.25E-10 | 212937 | 1.75E-02 | 380 |
| rs116758471 | G | 33602897 | 0.04 | 0.72 | 0.069 | 1.84E-25 | 212937 | 4.10E-02 | 910 |
| rs13093110 | T | 188125120 | 0.54 | 0.222 | 0.034 | 8.83E-11 | 212937 | 2.45E-02 | 534 |
| rs1611236 | A | 29748690 | 0.22 | 0.39 | 0.041 | 8.04E-22 | 212937 | 5.28E-02 | 1187 |
| rs3134971 | C | 32654149 | 0.10 | 1.862 | 0.057 | 1.00E-200 | 212937 | 6.32E-01 | 36518 |
| rs3916365 | G | 29913969 | 0.11 | -0.319 | 0.056 | 1.52E-08 | 212937 | 1.97E-02 | 427 |
| rs62404122 | C | 32614857 | 0.09 | -0.701 | 0.059 | 3.97E-32 | 212937 | 7.90E-02 | 1825 |
| rs653178a | T | 112007756 | 0.58 | -0.204 | 0.034 | 3.09E-09 | 212937 | 2.02E-02 | 440 |
| rs71542418 | C | 32587994 | 0.09 | -0.695 | 0.059 | 1.24E-31 | 212937 | 7.71E-02 | 1779 |
| rs9264277a | C | 31224667 | 0.73 | 0.463 | 0.04 | 4.10E-31 | 212937 | 8.45E-02 | 1965 |
| rs9265890 | A | 31313972 | 0.06 | 2.106 | 0.088 | 1.47E-126 | 212937 | 4.75E-01 | 19273 |
| rs9296068a | G | 32988695 | 0.44 | 0.548 | 0.035 | 4.17E-54 | 212937 | 1.48E-01 | 3685 |
| AIH | | | | | | | | | |
| SNP | EA | Position | EAF | BETA | SE | P | N | R^2^ | F |
| rs179247 | G | 81432546 | 0.58 | -0.373 | 0.047 | 2.47E-15 | 173938 | 6.80E-02 | 2114 |
| rs6679677 | A | 114303808 | 0.14 | 0.469 | 0.069 | 1.20E-11 | 173938 | 5.30E-02 | 1623 |
| rs72891915 | A | 33476200 | 0.04 | 0.685 | 0.12 | 1.31E-08 | 173938 | 3.54E-02 | 1063 |
| rs9265890a | A | 31313972 | 0.05 | 1.158 | 0.139 | 8.22E-17 | 173938 | 1.37E-01 | 4582 |
| rs9271671 | T | 32592908 | 0.58 | -0.312 | 0.051 | 8.19E-10 | 173938 | 4.75E-02 | 1446 |
| rs9275576 | T | 32679326 | 0.09 | 0.953 | 0.086 | 1.70E-28 | 173938 | 1.56E-01 | 5345 |
| rs942495 | T | 33798580 | 0.07 | 0.502 | 0.09 | 2.75E-08 | 173938 | 3.33E-02 | 999 |
| CD | | | | | | | | | |
| SNP | EA | Position | EAF | BETA | SE | P | N | R^2^ | F |
| rs13194642 | T | 32211695 | 0.08 | 0.485 | 0.088 | 3.16E-08 | 218792 | 3.59E-02 | 2035 |
| rs34022406 | C | 5350668 | 0.04 | 0.826 | 0.126 | 6.44E-11 | 218792 | 5.60E-02 | 3243 |
| rs62443225 | A | 5482137 | 0.08 | 0.638 | 0.094 | 9.56E-12 | 218792 | 5.95E-02 | 3459 |
| rs76176364 | G | 50519742 | 0.02 | 1.243 | 0.19 | 5.69E-11 | 218792 | 6.00E-02 | 3493 |
| UC | | | | | | | | | |
| SNP | EA | Position | EAF | BETA | SE | P | N | R^2^ | F |
| rs10737481 | G | 20171514 | 0.51 | 0.157 | 0.023 | 2.76E-12 | 214620 | 1.24E-02 | 538 |
| rs10807943 | C | 5340664 | 0.94 | -0.367 | 0.048 | 2.78E-14 | 214620 | 1.59E-02 | 695 |
| rs12139150a | C | 161470505 | 0.44 | -0.125 | 0.023 | 3.50E-08 | 214620 | 7.77E-03 | 336 |
| rs12946510 | T | 37912377 | 0.52 | 0.127 | 0.023 | 1.73E-08 | 214620 | 8.08E-03 | 350 |
| rs1391371a | T | 32603798 | 0.19 | -0.2 | 0.029 | 6.49E-12 | 214620 | 1.25E-02 | 545 |
| rs1391372a | C | 32604039 | 0.24 | -0.195 | 0.027 | 9.07E-13 | 214620 | 1.39E-02 | 603 |
| rs3197999 | A | 49721532 | 0.39 | 0.146 | 0.023 | 3.14E-10 | 214620 | 1.01E-02 | 438 |
| rs4676410 | A | 241563739 | 0.27 | 0.156 | 0.026 | 9.07E-10 | 214620 | 9.63E-03 | 417 |
| Psoriasis | | | | | | | | | |
| SNP | EA | Position | EAF | BETA | SE | P | N | R^2^ | F |
| rs10829130 | A | 27174346 | 0.11 | 0.197 | 0.036 | 4.24E-08 | 216752 | 7.56E-03 | 183 |
| rs12188300a | T | 158829527 | 0.06 | 0.433 | 0.05 | 2.24E-18 | 216752 | 2.08E-02 | 512 |
| rs12713428 | C | 61118113 | 0.25 | 0.169 | 0.026 | 8.11E-11 | 216752 | 1.07E-02 | 261 |
| rs13210419 | A | 31266977 | 0.06 | 1.116 | 0.051 | 1.10E-105 | 216752 | 1.37E-01 | 3833 |
| rs17728338 | A | 150478318 | 0.07 | 0.309 | 0.044 | 1.76E-12 | 216752 | 1.30E-02 | 317 |
| rs2021511 | T | 11344903 | 0.27 | -0.139 | 0.025 | 4.75E-08 | 216752 | 7.52E-03 | 182 |
| rs28752856a | G | 31298421 | 0.11 | 0.833 | 0.039 | 5.90E-100 | 216752 | 1.37E-01 | 3807 |
| rs28998802 | A | 26124908 | 0.19 | 0.167 | 0.029 | 7.41E-09 | 216752 | 8.43E-03 | 205 |
| rs4713605a | A | 32985992 | 0.33 | 0.153 | 0.024 | 2.35E-10 | 216752 | 1.03E-02 | 251 |
| rs60600003 | G | 37382465 | 0.10 | 0.213 | 0.037 | 1.03E-08 | 216752 | 8.27E-03 | 201 |
| rs674451 | C | 138216788 | 0.34 | 0.131 | 0.024 | 2.82E-08 | 216752 | 7.70E-03 | 187 |
| rs9481169 | T | 111929862 | 0.08 | 0.252 | 0.042 | 2.47E-09 | 216752 | 9.12E-03 | 222 |
| SS | | | | | | | | | |
| SNP | EA | Position | EAF | BETA | SE | P | N | R^2^ | F |
| rs2853986 | C | 31338844 | 0.09 | 0.775 | 0.074 | 1.43E-25 | 214435 | 1.01E-01 | 12057 |
| rs35407265 | G | 32591226 | 0.12 | -0.369 | 0.061 | 1.79E-09 | 214435 | 2.94E-02 | 3244 |
| rs3778754a | G | 128575552 | 0.43 | 0.283 | 0.041 | 3.16E-12 | 214435 | 3.94E-02 | 4393 |
| rs9265957a | T | 31317850 | 0.09 | 0.696 | 0.073 | 1.37E-21 | 214435 | 8.23E-02 | 9620 |
| SLE | | | | | | | | | |
| SNP | EA | Position | EAF | BETA | SE | P | N | R^2^ | F |
| rs41272536 | G | 183440531 | 0.04 | 1.132 | 0.181 | 4.05E-10 | 213683 | 9.35E-02 | 7342 |
| rs4713573 | C | 32626984 | 0.40 | 0.528 | 0.067 | 4.60E-15 | 213683 | 1.34E-01 | 10991 |
| rs9273324 | T | 32623156 | 0.20 | 0.648 | 0.083 | 6.00E-15 | 213683 | 1.33E-01 | 10933 |

IMIDs, immune-mediated inflammatory diseases; SNP, single-nucleotide polymorphism; EAF, effect allele frequency; EA, effect allele; BETA, beta. exposure; SE, standard error; P, the Significance level of immune-mediated inflammatory diseases; R² was calculated as follows: 2*BETA^2*EAF*(1-EAF). The F-statistic for each SNP was calculated as follows: F =( (N-K-1) /K)*(R^2^/ (1− R^2^)), K refer to the number of SNP , a Palindromic SNPs with intermediate allele frequencies (>0.01) were removed, RA, Rheumatoid arthritis; T1D, Type 1 diabetes; CeD, Coeliac disease; AIH, Autoimmune hyperthyroidism; CD, Crohn's disease; UC, Ulcerative colitis; SS, Sicca syndrome; SLE, Systemic lupus erythematosus.
