# Supplementary material for: Gene association analysis to determine the causal relationship between immune-mediated inflammatory diseases and frozen shoulder
Source: Medicine (Baltimore). 2024 May 10;103(19):e38055. doi: 10.1097/MD.0000000000038055 (PMC11081594; doi:10.1097/MD.0000000000038055)
Supplement: Supplementary file 3 [file medi-103-e38055-s008.docx]

**Supplementary Table 3** | Characteristics of SNPs associated with frozen shoulder.

| SNP | EA | Position | BETA | SE | P | N | R^2^ | F |
| --- | --- | --- | --- | --- | --- | --- | --- | --- |
| rs1042704 | A | 23312594 | 0.103 | 0.017 | 1.10E-09 | 451099 | 8.14E-05 | 9 |
| rs117999064 | G | 39977076 | 0.304 | 0.055 | 4.20E-08 | 451099 | 6.77E-05 | 8 |
| rs5777216 | G | 115681565 | -0.087 | 0.014 | 1.10E-09 | 451099 | 8.56E-05 | 10 |
| rs62228068 | T | 46384477 | 0.184 | 0.017 | 9.50E-29 | 451099 | 2.60E-04 | 29 |

SNP, single-nucleotide polymorphism; EAF, effect allele frequency; EA, effect allele; BETA, beta. exposure; SE, standard error; P, the Significance level of IMIDs; R² was calculated as follows: BETA^2/(BETA^2+SE^2*N). The F-statistic for each SNP was calculated as follows: F =( (N-K-1) /K)*(R^2^/ (1− R^2^)), K refer to the number of SNP.
