# Supplementary material for: Gene association analysis to determine the causal relationship between immune-mediated inflammatory diseases and frozen shoulder
Source: Medicine (Baltimore). 2024 May 10;103(19):e38055. doi: 10.1097/MD.0000000000038055 (PMC11081594; doi:10.1097/MD.0000000000038055)
Supplement: Supplementary file 4 [file medi-103-e38055-s009.docx]

**Supplementary Table 4** | Heterogeneity and pleiotropy analysis in reverse MR analysis.

| **Outcome** | **MR Method** | **Cochran Q statistic** | **Egger intercept** | **Heterogeneity p_value** | **Pleiotropy p_value** |
| --- | --- | --- | --- | --- | --- |
| **RA** | MR Egger | 7.45 | 0.008 | 0.682 | 0.309 |
|  | IVW | 8.60 |  | 0.658 |  |
| **T1D** | MR Egger | 11.52 | -0.041 | 0.210 | 0.298 |
|  | IVW | 15.63 |  | 0.080 |  |
| **Hypothyroidism** | MR Egger | 9.64 | -0.001 | 0.473 | 0.935 |
|  | IVW | 9.65 |  | 0.562 |  |
| **CeD** | MR Egger | 30.49 | -0.016 | 0.083 | 0.114 |
|  | IVW | 34.44 |  | 0.054 |  |
| **AIH** | MR Egger | 10.60 | -0.006 | 0.225 | 0.572 |
|  | IVW | 11.06 |  | 0.271 |  |
| **CD** | MR Egger | 1.87 | 0.010 | 0.393 | 0.851 |
|  | IVW | 1.91 |  | 0.591 |  |
| **UC** | MR Egger | 0.43 | -0.020 | 0.934 | 0.365 |
|  | IVW | 1.57 |  | 0.815 |  |
| **Psoriasis** | MR Egger | 8.49 | 0.001 | 0.292 | 0.949 |
|  | IVW | 8.49 |  | 0.387 |  |
| **SS** | IVW | 2.08 | NA | 0.149 | NA |
| **SLE** | MR Egger | <0.01 | 0.041 | 0.965 | 0.495 |
|  | IVW | 1.03 |  | 0.596 |  |

MR,mendelian randomization; IVW, Inverse variance weighted, RA, Rheumatoid arthritis; T1D, Type 1 diabetes; CeD, Coeliac disease; AIH, Autoimmune hyperthyroidism; CD, Crohn's disease; UC, Ulcerative colitis; SS, Sicca syndrome; SLE, Systemic lupus erythematosus;
